# Metabolite Profiling of Premium Civet Luwak Bio-Transformed Coffee Compared with Conventional Coffee Types, as Analyzed Using Chemometric Tools

**DOI:** 10.3390/metabo13020173

**Published:** 2023-01-24

**Authors:** Mohamed A. Farag, Tarik A. Mohamed, Enas A. El-Hawary, Amr Abdelwareth

**Affiliations:** 1 Pharmacognosy Department, College of Pharmacy, Cairo University, Kasr El-Aini St., Cairo 11562, Egypt; 2 Chemistry of Medicinal Plants Department, National Research Centre, 33 El-Bohouth St., Giza 12622, Egypt; 3 Chemistry Department, School of Sciences & Engineering, The American University in Cairo, New Cairo 11835, Egypt; 4 Novartis Pharma, Cairo Site, El-Sawah St., Cairo 11551, Egypt

**Keywords:** Copi-Luwak, coffee, chemometrics, NMR, GC-MS, UPLC-MS

## Abstract

Luwak (civet) coffee is one of the most precious and exotic coffee commodities in the world. It has garnered an increasing reputation as the rarest and most expensive coffee, with an annual production. Many targeted analytical techniques have been reported for the discrimination of specialty coffee commodities, such as Luwak coffee, from other ordinary coffee. This study presents the first comparative metabolomics approach for Luwak coffee analysis compared to other coffee products, targeting secondary and aroma metabolites using nuclear magnetic resonance (NMR), gas chromatography (GC), or liquid chromatography (LC) coupled with mass spectrometry (MS). Chemometric modeling of these datasets showed significant classification among all samples and aided in identifying potential novel markers for Luwak coffee from other coffee samples. Markers have indicated that *C. arabica* was the source of Luwak coffee, with several new markers being identified, including kahweol, chlorogenic acid lactones, and elaidic acid. Aroma profiling using solid-phase micro-extraction (SPME) coupled with GC/MS revealed higher levels of guaiacol derivatives, pyrazines, and furans in roasted Luwak coffee compared with roasted *C. arabica*. Quantification of the major metabolites was attempted using NMR for Luwak coffee to enable future standardization. Lower levels of alkaloids (caffeine 2.85 µg/mg, trigonelline 0.14 µg/mg, and xanthine 0.03 µg/mg) were detected, compared with *C. arabica*. Other metabolites that were quantified in civet coffee included kahweol and difurfuryl ether at 1.37 and 0.15 µg/mg, respectively.

## 1. Introduction

Luwak (civet) coffee is one of the most precious exotic coffee commodities traded in the world [1]. It has garnered an increasing reputation as the rarest and most expensive coffee, with an annual production of around 500 pounds and 600 dollars per pound, which is approximately one hundred times that of normal coffee [2]. Such a high price is attributed to its high consumer demand and superior sensory attributes. Luwak coffee is low in caffeine, low in fat, and low in bitterness. A study of different samples of civet coffee from the Gayo Highlands showed that nutty, fishy, chocolaty, herby, toasty, and earthy flavors were the dominant characteristics [3]. *Coffea arabica* berries are first digested by the Asian palm civet (*Paradoxurus hermaphroditus*), then the stools are either collected in the wild or harvested from caged animals. This arboreal animal is an excellent tree climber; it uses its strong sense of smell and eyesight to select only the ripest and sweetest coffee cherries [4]. The civet digests the berries’ pericarp and ejects coffee beans that then undergo cleaning, wet fermentation, sun-drying, and, finally, roasting [1]. Throughout the fermentation process, a unique flavor is imparted to the coffee beans that has a chocolaty, earthy, syrupy, and rich taste [4].

Indonesia is the first and main producer of Luwak coffee; it became more popular in 2003 when Oprah Winfrey presented Luwak coffee in her TV show [3]. Luwak coffee can be produced naturally in the wild, or the animals can be kept in cages, which is referred to as captivity. Studies have reported that good breeding methods and gentle treatment for the animals can lead to an increase in the production of premium-quality caged coffee, compared to naturally produced wild coffee [5]. The use of caged-animal breeding was developed to meet the increasing demand for civet coffee due to the scarcity of wild Luwak coffee horns. However, this approach is challenged by the need to maintain animal nutrition availability and healthy caged production, which is necessary for caged civet-produced coffee that approaches the sensory quality of wild civet-produced coffee [3].

Civet coffee beans are characterized by less bright, darker coffee beans compared with non-fermented coffee beans [3]. Studies have focused more on developing an analytical process to authenticate Luwak coffee and detect adulteration by various means, including the sensory evaluation of green coffee beans and instrumental nose and olfactory analysis, culminating with the employment of large-scale metabolomics to identify discriminatory markers [1].

According to many studies, the “better” taste of Luwak coffee has been attributed to the natural fermentation process, which contributes to the production of several flavors that give it a special aroma [5]. Fermentation is performed using the gut microbiota and enzymes in the Luwak gastrointestinal tract. Sugar, protein, and pectin are degraded into simpler forms with the aid of digestive enzymes; studies have, moreover, isolated the microbial strains in the biomass from palm civets, which are responsible for the catabolism of caffeine in the coffee bulb via *N*-demethylation and xanthine oxidation [6]. Gluconobacter species possessing genes that encode enzymes for the metabolism of sulfur-containing amino acids have been reported in palm civet feces [7]. The degradation of structural polysaccharides eases the penetration of fermented metabolites into coffee beans. The presence of amino acid residues from fermentation contributes to the sensory notes of coffee and contributes to the enriched aroma post-Milliard reaction that occurs during coffee roasting [8]. Studies are now focusing on finding a starter consortium microbiota that mimics the in vivo fermentation occurring in the palm civet gut to manufacture Luwak coffee by artificial bio-fermentation on a commercial scale [3].

Several analytical techniques have been employed for the identification and quantification of major coffee metabolites, such as caffeine and trigonelline. These techniques have ranged from simple planar chromatography [9,10,11] to the use of more advanced techniques that are based on holistic metabolomics data analysis for the discrimination of specialty coffee commodities, including Luwak coffee, from other ordinary coffee-adulterant commodities. These include mass spectrometry (MS) coupled with chromatography, inductively coupled plasma atomic emission spectrometry (ICP-AES), Fourier transform infrared (FTIR) spectroscopy, Raman spectroscopy, nuclear magnetic resonance (NMR), and GC with FID detection [12].

**Figure 1:**
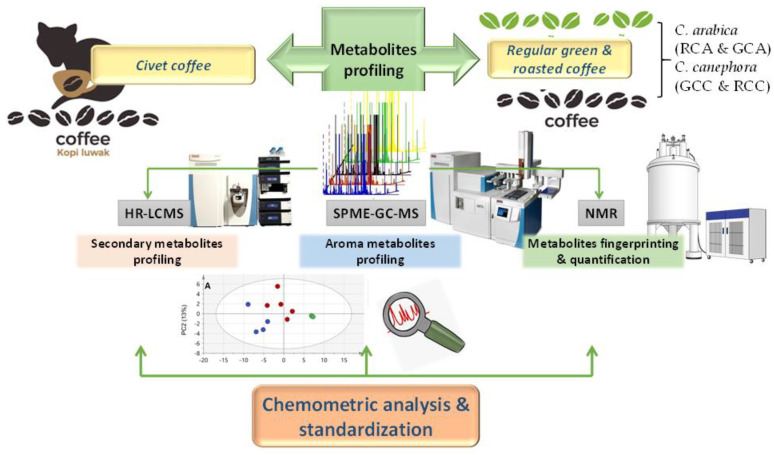
Graphical sketch summarizing the current paper’s objectives and the techniques employed for comparisons between Luwak and regular coffee types.

This paper is intended to complement our previous work on the different coffee species and commodities, using multiple analytical techniques for metabolite fingerprinting based on ultraviolet spectroscopy, liquid chromatography-mass spectrometry, silylated GC-MS, SPME/GC-MS, and NMR, all coupled with modeling using chemometric tools (see Figure 1) [13,14].

Recently, metabolomics has made a remarkable contribution to the analysis of food components and quality control. Several hyphenated techniques, such as nuclear magnetic resonance (NMR), gas chromatography (GC), or liquid chromatography (LC) coupled to mass spectrometry (MS), have been employed to analyze food phytochemicals and provide a detailed profile of their composition. Liquid chromatography is a powerful tool for investigating the differences in the chemical profile between closely related taxa and species, due to its excellent resolution and high sensitivity level. Most previous metabolomics studies have targeted the diverse commercial types of coffee and have provided a detailed profile of the different coffee species, i.e., *C. arabica, C. canephora, C. liberica*, etc. Although the unique coffee analyzed in this paper has attracted many coffee lovers and has increased in demand worldwide, there is little research into the identification of significant phytochemicals and discriminant markers for its authentication, compared to other coffee types. The objective of this study was to employ chemometric tools for the first time to assess the phytochemicals in Luwak coffee, targeting its aroma and secondary metabolites, and standardizing its major components as one of the priciest and rarest coffees available.

## 2. Materials and Methods

### 2.1. Coffee Specimens, Chemicals, and Extraction

Commercial Luwak coffee was purchased from Bogor Indonesia as 100% pure, coarsely powdered coffee, ca. 2–4 mm in size, which has been heavily roasted and freeze-dried. It was compared to samples from two coffee-producing species: *C. arabica*, commonly known as arabica coffee, roasted arabica coffee (RCA), and green arabica coffee (GCA); the other is *C. canephora* var. robusta (known as green robusta coffee (GCC) or roasted robusta coffee (RCC)), collected from the Mina Gerais University Arboretum, Brazil, as entire seeds that were further powdered in a mortar using liquid nitrogen. Analysis was performed via NMR spectroscopy, ultra-performance liquid chromatography coupled with mass spectroscopy (UPLC-MS), and solid-phase microextraction coupled with the gas chromatography-mass spectrometry method (SPME/GC–MS). Samples subjected to SPME/GC-MS included Luwak coffee, roasted coffee, and roasted coffee blended with cardamom to comparatively evaluate the aroma profile. The NMR fingerprinting of coffee extracts was also conducted.

Freeze-dried coffee seeds were prepared for NMR analysis following the same protocol as used for herbal extracts [15,16,17]; about 150 mg of each coffee powder (*n* = 3) was homogenized with 6 mL of 100% MeOH containing 10 µg/mL umbelliferone (an internal standard for relative quantification using LC-MS), using an Ultra-Turrax (IKA, Staufen, Germany) at 11,000 rpm for 5 × 60 s, with 1-minute break intervals. The extract was vortexed for 1 min, centrifuged at 3000× *g* for 30 min, and then filtered. Afterward, 4 mL of the supernatant was aliquoted for NMR analysis and then dried in a stream of nitrogen. The dried extract was re-suspended with 800 μL of 100% methanol-d4, containing HMDS that has been adjusted to a final concentration of 0.94 mM. After centrifugation (13,000× *g* for 1 min), the supernatant was transferred to a 5-millimeter NMR tube for measurement. For the LC-MS analysis, 1 mL of the sample was aliquoted and placed on a 500 mg Octadecylsilane (C18) cartridge that was preconditioned with methanol. The samples were then eluted using 3 × 0.5 mL methanol; the eluent was then evaporated under a nitrogen stream and the obtained dry residue was resuspended in 1 mL of 100% methanol.

### 2.2. UPLC-MS Profiling of Secondary Metabolites

UPLC-MS acquisition was performed using ion-trap high-resolution testing under the same conditions as those used for coffee testing by El-Hawary et al. [13].

To profile the metabolites, 150 mg of each coffee powder specimen was homogenized with 5 mL MeOH (100% *v/v*) containing 10 µg/mL umbelliferone as an internal standard, using an Ultra-Turrax mixer (IKA, Staufen, Germany) adjusted at 11,000 rpm, mixed in five 20-second periods, with intervals of 1 min between each mixing period to guard against temperature increases and heating effects. The resulting suspensions were then vortexed vigorously, centrifuged at 3000× *g* for 30 min, and filtered through a 22 μm pore-size filter to remove plant debris. Then, 1 mL of the sample was aliquoted and pre-treated by placement on a 500 mg C_18_ cartridge that was pre-conditioned with MeOH and Milli-Q water before elution; this was performed twice, using 3 mL of MeOH. Afterward, the eluent was evaporated under a nitrogen stream, and the obtained dry residue was re-suspended in 1 mL of MeOH.

The principal step of UPLC-ESI–HRMS analysis was conducted in triplicate (*n* = 3), with 2 μL introduced to a Dionex 3000 UPLC system (Thermo Fisher Scientific, Bremen, Germany), equipped with an HSS T3 column (100 × 1.0 mm, 1.8 μm; Waters^®^; column temperature: 40 °C) and a photodiode array detector (PDA, Thermo Fisher Scientific, Bremen). The chromatographic conditions were optimized for improved peak elution, using a binary gradient elution protocol at a flow rate of 150 μL/min. The composition of the mobile phase varied between water/formic acid at 99.9/0.1 (*v/v*) (A) and acetonitrile/formic acid at 99.9/0.1 (*v/v*) (B). The protocol consisted of an isocratic step for 1 min with 5% mobile phase B, followed by a linear increase of B from 5% to 100% over 11 min. The mobile phase was kept isocratic for between 11 and 19 min at 100% B. After this, there was a return to 5% B within 1 min, and, finally, an additional 10 min, i.e., 20–30 min overall, for column re-equilibration using 5% B. The wavelength range of the PDA measurements used for detection was 190–600 nm.

The UPLC system was coupled with a high-resolution mass spectrometer, comprising an Orbitrap Elite mass spectrometer (Thermo Fisher Scientific, Bremen, Germany) equipped with a HESI electrospray ion source (spray voltage, positive ion mode 4 kV, negative ion mode 3 kV; source heater temperature, 250 °C; capillary temperature, 300 °C; FTMS resolution, 30,000). Nitrogen was used as both the sheath and auxiliary gas. The CID mass spectra (buffer gas: helium; FTMS resolution: 15,000) were recorded in a data-dependent acquisition mode (DDA) using normalized collision energy (NCE) of 35% and 45% The instrument was externally calibrated with Pierce^®^ LTQ Velos ESI positive ion calibration solution (product number 88323, Thermo Fisher Scientific, Rockford, IL, USA) and Pierce^®^ LTQ Velos ESI negative ion calibration solution (product number 88324, Thermo Fisher Scientific, Rockford, IL, USA).

### 2.3. Headspace SPME GC-MS Profiling of Aroma Compounds

GC/MS analysis of coffee volatiles was performed exactly as previously described by Farag et. al. [17,18]. Three biological replicates were analyzed for each specimen, using a Shimadzu GC-17A gas chromatograph equipped with a DB-5 column (30 m, 0.25 mm × 0.25 um film thickness; Supelco^®,^ Merck SA, Darmstadt, Germany) coupled to a Shimadzu QP5050A mass spectrometer.

### 2.4. NMR Fingerprinting of Coffee Extracts

All spectra were recorded using an Agilent VNMRS 600 NMR spectrometer (Varian, Palo Alto, CA, USA) at a proton NMR frequency of 599.83 MHz, using a 5-millimeter inverse detection cryoprobe. The ^1^H-NMR spectra were recorded at the parameters, including a digital resolution of 0.367 Hz/point, pulse width (pw) of 3 μs (90°), relaxation delay of 23.7 s, and an acquisition time of 2.7 s; the number of transients was 160. Zero filling up to 128 K (l b = 0.4) was used prior to the Fourier transformation. 

### 2.5. Data Processing and Multivariate Analysis 

#### 2.5.1. NMR Quantification of Coffee Metabolites and Dataset Modeling

The NMR spectra were processed with Mestrenova software (Mestrelab Research Mnova 14.1.0 Build 24037) to aid in the peak picking (*δ*_H_, *δ*_C_, and *δ*_H/C_) of detected NMR signals, measured in parts per million (ppm) relative to the internal standard hexamethyldisilazane (HMDS). In addition, the ^1^H-NMR spectra were automatically Fourier-transformed to ESP files using the ACD/NMR Manager Lab version 10.0 software (Toronto, Canada), based on the method used by [15,16]. 

Further processing was applied for multivariate analysis (MVA), including the spectra binning into buckets of equal width (0.04 ppm) within the region of *δ*_H_ at 11.4–0.4 ppm and the exclusion of signals between *δ*_H_ at 5.0–4.7 ppm and *δ*_H_ at 3.4–3.25 ppm, corresponding to the residual water and methanol signals, respectively. The data were then subjected to principal component analysis (PCA), hierarchical component analysis (HCA), and orthogonal partial least-squares discriminant analysis (OPLS-DA) using the SIMCA-P version 14.1 software package (Umetrics, Umeå, Sweden). All variables were mean-centered and scaled to the Pareto variance. The models were derived from both the full scale of the chemical shift (*δ*_H_: 1–10 ppm) and the aromatic region (*δ*_H_: 5.4–10 ppm). Quantification followed the exact formulae and procedures described in earlier works [15,16].

#### 2.5.2. SPME-GC/MS Dataset Volatiles Identification and Modeling 

Volatiles were identified by the comparison of peak retention time, the Kovat index (KI), and the spectrum with the reference metabolites in the NIST database (NIST/EPA/NIH mass spectral database (NIST 11). For peak identification, peaks were first deconvoluted using the AMDIS software (www.amdis.net) (accessed on 1 December 2022), prior to spectral matching. The relative content of each metabolite was obtained by the area normalization of all responses related to the identified hits. Average responses per injection replicates were then calculated for each metabolite. Afterward, the data were subjected to multivariate analysis (MVA), as described earlier, with the NMR dataset deriving models from the full scale of both the chemical shift and the aromatic region.

#### 2.5.3. UPLC-ESI–HRMS Dataset Metabolite Identification and Modeling 

All metabolites were identified by their accurate mass, retention time, MS fragments, isotopic distribution, and error. The “*X*-caliber software qual” browser (https://www.thermofisher.com/)(accessed on 1 December 2022) was used for the imported high-resolution files. The analysis was performed in negative mode, and the ion mass spectra that were derived from the anions (M−H) were accompanied by many fragmentation patterns. Relative comparisons of the spectral data were made with the literature references, in-house data, and natural products database of the standard phytochemical dictionary (CRC, Wiley).

The original LC-MS files of all authenticated samples (GCC, GCA, RCA, and RCC) and the Luwak samples were converted into mzML files using the MS Convert GUI (http://proteowizard.sourceforge.net/download.html) (accessed on 1 December 2022) and then converted to .abf files using the ABF converter (https://www.reifycs.com/AbfConverter/) (accessed on 1 December 2022), with the exact parameters described in our previous study [13]. The peak abundance mass list was then exported for multivariate data analysis, wherein the final ID and metabolites were Pareto-scaled using SIMCA (Umetrics, Umea, Sweden). The unsupervised principal component analysis (PCA) models were validated, based on R and Q, in addition to a hierarchal cluster analysis (HCA) of the authenticated and Luwak samples. Supervised OPLS-DA analysis was used in the pre-classified groups to identify the requisite markers, via an S-plot that was validated using the *p*-value, covariance (*p*), and correlation (pcor). 

## 3. Results

### 3.1. H-NMR Assignments and the Quantification of Coffee Metabolites

#### 3.1.1. Identification of Coffee Metabolites 

^1^H-NMR analysis was employed for Luwak coffee characterization and the quantification of its major peaks, to be used for its future standardization (Table 1). 

**Figure 2:**
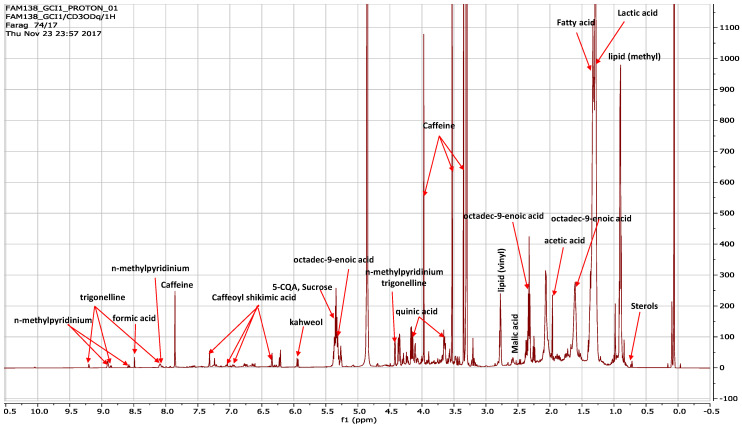
Representative ^1^H NMR spectrum (*δ*_H_: 0–10 ppm) of the Luwak coffee sample in CD_3_OD.

The representative 1D ^1^H-NMR spectra are depicted in Figure 2. The Luwak coffee sample displayed a signal richness that can mostly be ascribed to primary metabolites found in the aliphatic region from 0 to 5 ppm, and the lower intensity ascribed to secondary metabolites found in the region from 5.5 to 10 ppm. Metabolites that were identified from both ranges included several major coffee metabolites, i.e., caffeine, trigonelline, *N*-methylpyridinum, kahweol, sucrose, caffeoyl shikimic acid, quinic acid, malic acid, lactic acid, acetic acid, sterols, and fatty acids. Considering that coffee’s health-promoting effects are mostly ascribed to its secondary metabolites, assignments of its key metabolites were attempted based on one- and two-dimensional NMR experiments, such as heteronuclear single-quantum correlation spectroscopy (HSQC), heteronuclear multiple-bond coherence (HMBC), etc.

With regard to the secondary bioactives, the ^1^H-NMR spectrum was characterized by dense signals in the mid-spectrum region (*δ*_H_ 3.4–4 ppm) belonging to caffeine, which was detected at *δ*_H_ 7.58 (H5), and three methyl signals at *δ*_H_ 3.34, 3.52, and 3.97 for CH_3_-8, CH_3_-7, and CH_3_-6, respectively [19]. Lower-intensity NMR signals were observed for another alkaloid, i.e., trigonelline annotated from singlet signals at *δ*_H_ 4.42 [20], owing to the *N*-methyl group. The trigonelline structure was confirmed, based on multiple signals at *δ*_H_ 8.0–9.5 ppm, which can be attributed to the aromatic protons. Due to its structural similarity to trigonelline, *N*-methylpyridinium showed overlap signals at *δ*_H_ 4.4 ppm and 8–9 ppm, owing to the *N*-methyl group and aromatic protons, respectively [21]. *N*-methylpyridinium (NMP) is a thermal degradation product of trigonelline and is hypothesized to exert several health benefits in humans [22]; it is likely to be generated during the roasting process of Luwak coffee. Dark-roasted coffee that is rich in NMP was shown to reduce body weight [23] and has yet to be examined in the case of Luwak coffee.

Kahweol is a diterpene that is reported as a marker of *C. arabica*, identified based on its two key doublet signals at *δ*_H_ 5.9 ppm (due to H-2), *δ*_H_ 6.2 ppm (due to H-1 and H-18), and *δ*_H_ 7.2 ppm (due to H-1 and H-19) [19]. The characterization of its signals verified that *C. arabica* was the origin material of Luwak coffee production in this product; an analysis of other Luwak coffee samples from other origins can further confirm such a hypothesis.

With regard to primary metabolites that contribute more to coffee’s sensory and nutritive attributes, malic acid was identified as the major organic acid to be characterized, based on a multiplet at *δ*_H_ 2.8 ppm, while the high-field region up to *δ*_H_ 2.0 ppm showed lactic acid and acetic acid signals [24]. Furthermore, signals characteristic of free sugars that might account for the taste of the coffee could be readily assigned in the ^1^H-NMR spectrum, including the anomeric proton of sucrose at *δ*_H_ 5.39 (d, *J* = 5.0 Hz) [17].

Organic acids, such as quinic acid, were identified at *δ*_H_ 3.57 (dd, *J* = 9.5, 3.2 Hz) and 4.14 (dd, *J* = 10.8, 6.4) ppm [25] as the major acids that contribute to the production of coffee key phenolics i.e., chlorogenic acid, the major antioxidant in coffee. Other phenolic acid derivatives were identified downstream of quinic acid, due to the acetylation of an acid moiety, including caffeoyl quinic acid (chlorogenic acid), which appeared at *δ*_H_ 5.32, owing to the presence of 5-CQA H10 [26], as identified in Figure 2. Caffeoyl shikimic acid signals were detected at δH 6.34 and 7.23 ppm, which correspond to H-4 and H-6, respectively [27]. 

Generally, the *δ*_H_ 0–3 ppm region showed considerably higher-intensity signals that are typical for organic/fatty acids and sterols [19]. Few of the signals characteristic of fatty acids could be readily assigned in the ^1^H-NMR spectrum, such as at *δ*_H_ 1.28 and 1.32 ppm, for the repeated methylene groups of fatty acids [19].

To overcome the signal overlap observed in the 1D-NMR spectra, a set of 2D-NMR spectroscopic experiments were employed for the assignment of coffee metabolites. The unsaturation in some fatty acid chains, as in the case of octadec-9-enoic acid (elaidic acid) was confirmed by the presence of a triplet at *δ*_H/C_ (1H, 5.35/129, t, *J* = 5.0 Hz), showing the HSQC cross-peak correlation to the aliphatic methylene (C-8, C-11) at *δ*_C_ 27.5 ppm. The annotation of free fatty acid was based on its carbonyl at *δ*_C_ 176 and the adjacent α-methylene at *δ*_H/C_ 2.38/35.0 and was consistent with that reported in [28] (see Figure S1C,D in the Supplementary Materials).

Elaidic acid is the trans-unsaturated fatty acid isomer of oleic acid. It has been regarded as detrimental to the sensory quality of coffee [29] and is generated during the thermal processing of coffee beans, leading to the transformation of fatty acids from a *cis* configuration to a trans configuration. Generally, elaidic acid is detected at low levels in coffee beans; being detected in Luwak coffee by the NMR indicates the effect of the roasting post-fermentation step. Although linoleic acid has been characterized via NMR in several studies, this is the first report to characterize elaidic acid in Luwak coffee beans.

#### 3.1.2. NMR Metabolites Quantification 

Quantification was further employed for the key major coffee chemicals, based on ^1^H-NMR, including alkaloids and nitrogenous compounds (i.e., caffeine, trigonelline, *N*-methylpyridinium, and xanthine), diterpene (i.e., kahweol), and phenolics (i.e., caffeoyl shikimic acid, octadec-9-enoic (elaidic) acid, and difurfuryl ether). 

Caffeine comprised the main alkaloid detected at 2.85 µg/mg (compared to 12.2 µg/mg and 11.1 µg/mg in GCA and RCA respectively), whereas trigonelline was detected at much lower levels of 0.14 µg/mg (compared to 10.6 µg/mg and 7.2 µg/mg in GCA and RCA, respectively), which is likely attributable to the roasting of Luwak coffee seeds during the preparation of samples, it being thermolabile [19]. Xanthine, a caffeine derivative, was quantified at low levels of 0.03 µg/mg; whether this is derived from microbiota-mediated fermentation inside the animal gut is yet to be determined.

Difurfuryl ether is a furan that contributes a coffee-like, nutty, earthy, mushroom odor [30]; it was previously reported, using SPME/GC-MS, in roasted coffee [14]. Quantification was based on *δ*_H_ 7.57, yielding concentrations of 0.156 µg/mg.

The diterpene, kahweol, which is a marker of *C. arabica* species, was detected at 1.378 µg/mg. This was much less than that in authentic roasted *C. arabica* RCA (8.8 µg/mg) and its green counterpart, GCA (9.7 µg/mg). Kahweol was not detected in either roasted or green robusta coffee samples [19].

Marked levels of the fatty acid, octadec-9-enoic (elaidic) acid, were quantified at 18.53 µg/mg. It was previously characterized in green *C. arabica* samples [19], and, likewise, in the coffee pigments of *C. arabica*, confirming its origin in the Luwak coffee samples [31], alongside kahweol diterpene.

### 3.2. Metabolite Profiling via UPLC-ESI–HRMS

Kopi Luwak extract was subjected to UPLC-MS analysis, allowing the annotation of 24 metabolites, as listed in Table 2. The order of eluted metabolites followed that in our previous paper [13] on authenticated green and roasted coffees, including organic acids, phenolic acids (i.e., hydroxycinnamates, feruloyl, and coumaroyl derivatives), amino acids, and fatty acids. A list of identified compounds, along with their spectroscopic data, is shown in Table 2. The fragmentation patterns of the identified metabolites have been presented in previous reports [13,32,33,34].

### 3.3. SPME/GC-MS Analysis of Luwak Coffee Aroma

A powdered sample of civet coffee was subjected to head-space extraction, coupled with GC-MS for aroma profiling. The obtained chromatograms were evaluated in comparison with chromatograms obtained from previous samples of authentic roasted *C. arabica* (RCA) and *C. arabica* with cardamom as a major blended coffee type, analyzed using the same method [14]. The reason for choosing RCA for comparison is the common origin and similar processing of samples. This is because civet coffee is obtained from Luwak animal-feed coffee arabica cherries, as revealed by NMR and LCMS modeling using PCA and HCA, showing the close grouping between civet coffee samples and roasted *C. arabica* samples. Commercial coffee with cardamom was added, owing to the distinct pattern of volatile metabolites associated with cardamom supplementation, as outlined in our previous report [14], and to compare whether civet coffee has an improved aroma profile.

As listed in Table 3, 75 peaks belonging to 13 chemical groups were verified. The identified volatiles included alcohols (6), aldehydes (2), aliphatic hydrocarbons (4), aromatic hydrocarbons (2), esters (7), ether/oxide (5), furan/pyran (14), ketone (3), monoterpene hydrocarbon (6), phenolics (3), pyrazines (12), and sesquiterpene hydrocarbons (10).

The Luwak coffee’s SPME/GC-MS chromatogram showed a comparable aroma profile to that of RCA and *C. arabica* with cardamom in terms of furans, pyrazines, phenolics, aliphatic and aromatic hydrocarbons, certain alcohols (i.e., furfuryl alcohol and maltol), certain aldehydes (2-methoxy-4-methylbenzaldehyde). Other volatiles, including ethylene diacetate and butyryl lactone, have been detected only in the Luwak samples.

On the other hand, the Luwak samples showed no detection of terpene hydrocarbons, most of the esters (i.e., linalyl acetate, methyl geranate, myrcenyl acetate, terpinyl acetate, and decyl acetate) that are abundant in roasted coffee with cardamom, owing to the cardamom content. Regarding the relative abundance of volatiles in Luwak coffee samples, furans and pyrans were the major class (40.1%), followed by alcohols (14.68%), pyrazines (13.33%), and phenolics (12.89%). Other classes that were present at much lower levels included aromatic hydrocarbons (4.82%), esters (1.04%), and ether/oxide (1.08%). For classes with a relative abundance in the Luwak aroma, please refer to Figure S2 in the Supplementary Materials. Major aroma compounds included furfuryl alcohol (12.1%), 4-ethylguiacol (9.59%), difurfuryl ether (8.96%), furfuryl acetate (6.47%), and methylpyrazine (6.05%). 

As identified in Table 3, the pyrazines showed a high abundance in Luwak coffee, compared with roasted *C. arabica*, which was most likely generated during the thermal processing (Milliard reaction) of coffee. The higher amount detected in Luwak coffee, compared to other roasted *C. arabica* samples, is in accordance with a previous report indicating the impact of solid fermentation on the amino acid and sugar precursors of pyrazines such as phenylalanine, aspartic acid, and glutamic acid. Substantial levels of 2-methylpyrazine were detected, which indicated high levels of both glutamic acid and aspartic acid amino acid precursors in the Luwak coffee, prior to roasting [35].

In a similar way, a higher abundance of phenolics, such as 4-ethylguiacol (9.59%), 4-vinyl guaiacol (1.78%), and guaiacol (1.52%), which were only detected in Luwak coffee, indicated the impact of fermentation in the civet’s gut on the phenolic precursors of hydroxycinnamic acid, whereas the higher abundance of 4-ethylguiacol, compared with 4-vinylguiacol, indicated the thermal processing during dark roasting [35].

## 4. Discussion

### 4.1. H-NMR Multivariate Data Analysis of Luwak Coffee and Authenticated Green and Roasted Coffees

Similar NMR spectra were observed by a visual examination of the ^1^H-NMR spectra of Luwak coffee and then compared with authenticated green (GCA, GCC) and roasted coffee (RCA and RCC) samples [19], revealing to which type Luwak coffee is close in an untargeted manner using chemometric tools. A report on the exact chemical characterization of the NMR data of these coffee samples has previously been reported by our group [19].

**Figure 3:**
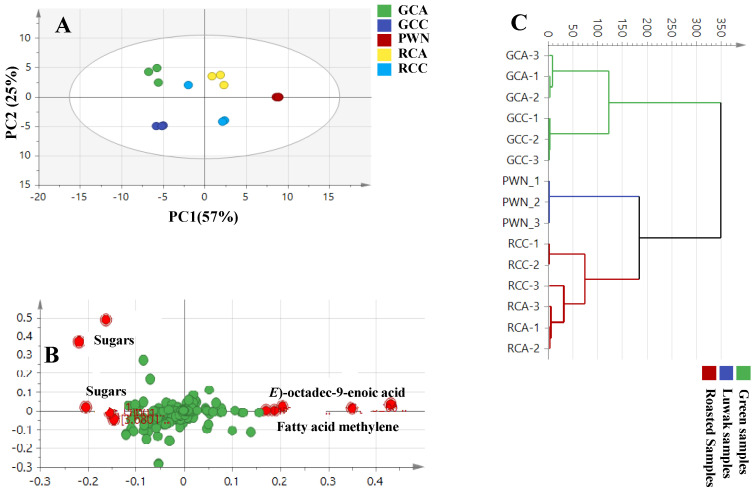

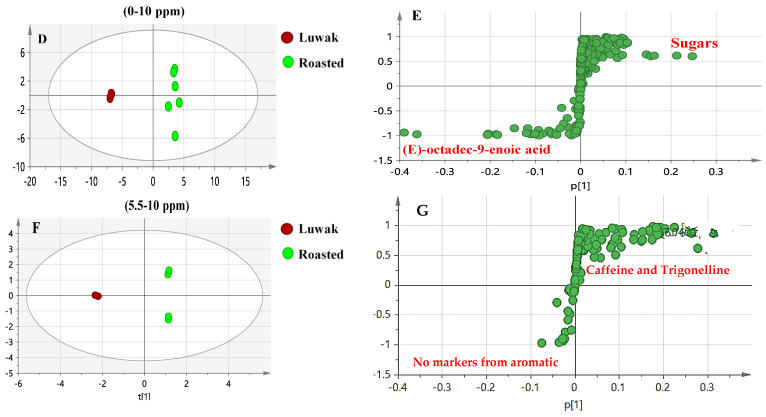
(**A**) The principal component analysis (PCA) score plot. (**B**) The loading plot of all the coffee samples, analyzed using NMR. (**C**) Hierarchical cluster analysis (HCA) plot. (**D**) OPLS-DA model for the roasted samples (RCA and RCC) vs. the Luwak coffee (PWN). (**E**) The S-loading plot showing the covariance *p* [1] against the correlation *p*(cor) [1], analyzed by 1 H NMR, shown at a full scale (**F**) OPLS-DA score plot for the roasted samples versus the roasted aromatic region. (**G**) The S-loading plot for the aromatic region. Selected variables are highlighted and discussed in the text.

The NMR-derived dataset was based on samples of authenticated green, roasted, and Luwak coffee, using both unsupervised and supervised analysis, as seen in Figure 3. The principal component analysis (PCA) plot showed the principal component, PC1, representing 57% and 25% of the PC2 of the total variance, with acceptable values for the goodness-of-fit and goodness-of-prediction (R^2^ = 0.57 and Q^2^ = 0.39), suggesting an acceptable model, as shown in Figure 3A. The corresponding loading plot revealed the enrichment of sugars in green coffee, while roasted and Luwak coffees were more abundant in fatty acids (see Figure 3B). HCA showed a similar segregation pattern (Figure 3C), in which samples were segregated into two main clusters. The first cluster included all green samples of both species; the Luwak coffee, along with all the roasted samples of both species (RCA and RCC), showed that the fatty acids were present in the second cluster, suggesting that the roasting process was more influential than the genotype among the full scans and aromatic models. 

To confirm the results revealed from the unsupervised PCA, another supervised OPLS-DA analysis of the full NMR (Figure 3D,E), and aromatic spectral regions (Figure 3F,G) was attempted, with good model parameters: R^2^ = 0.93, Q^2^ = 0.91, R^2^ = 0.83, Q^2^ = 0.80, and a *p*-value of less than 0.05 for the full coffee region at *δ*_H_: 0–10 ppm and 5.5–10 ppm, respectively. The full scan model (*δ*_H_: 0–10 ppm) provided better classification than the aromatic region (*δ*_H_: 5.5–10 ppm), based on these validation parameters. The OPLS S-loading plot (*δ*_H_: 0–10 ppm) confirmed the PCA results for the high abundance of fatty acids in Luwak coffee versus the enrichment of roasted coffee in sugars as the most discriminatory ^1^H-NMR signals (see Figure 3E). Lastly, an OPLS of the aromatic region’s ^1^H-NMR signals (*δ*_H_: 5.5–10 ppm) dataset showed a higher abundance of caffeine and trigonelline in the roasted samples of both species, whereas, interestingly, no markers were detected for Luwak (see Figure 3G).

### 4.2. UPLC–HRMS Multivariate Data Analysis of the Luwak Coffee and Authenticated Coffee Samples

**Figure 4:**
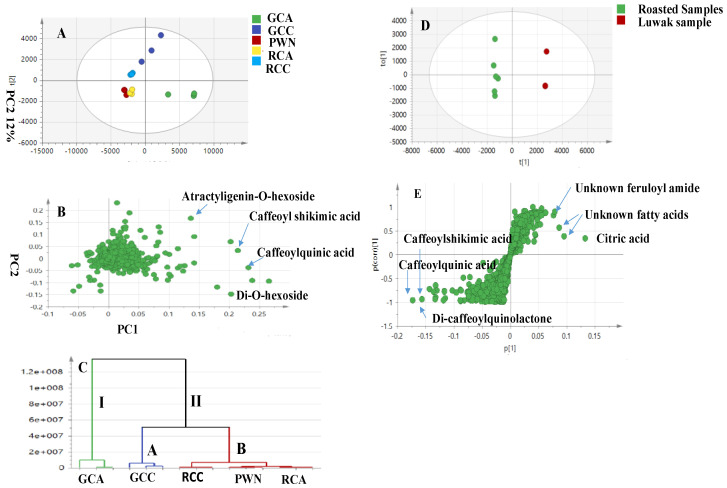
(**A**) Principal component analysis (PCA) score plot of PC1 vs. PC2 scores, based on the UPLC-MS of authentic samples (arabica and canephora specimens) and Luwak samples. (**B**) PCA loading plot for PC1 and PC2, showing the potential markers for roasted coffee seeds. (**C**) HCA of the authentic and Luwak samples. (**D**) OPLS-DA score plot for the Luwak versus the roasted samples only. (**E**) OPLS-DA S-plot for the Luwak versus the roasted samples that are contributing markers for Luwak coffee. OPLS-DA-S-plot models show the covariance-*p* [1] against the correlation *p*(cor) [1] for the variables of the discriminating components of the OPLS-DA models. Cut-off values of *p* < 0.05 were used; selected variables are highlighted in the S-plot and are discussed in the text.

The UPLC–HRMS dataset was classified using multivariate data analysis, including the previously characterized coffee samples (authenticated green and roasted coffee) and Luwak coffee, all analyzed using the same method [13]. Both unsupervised analyses, i.e., PCA and HCA, and supervised analyses, i.e., OPLS-DA, were constructed for specimen classification and for the identification of distinct markers for Luwak coffee (Figure 4).

Firstly, the PCA model, taken as an unsupervised model, was applied for five samples, including Luwak (PWN) and the authenticated green and roasted samples denoting different symbols, i.e., green canephora coffee (GCC), green *C. arabica* (GCA), roasted *C. arabica* (RCA), and roasted canephora coffee (RCC). The PCA score plot (Figure 4A) explained 48% of the total variance in PC1, whereas the second principal component, PC2, explained 12% of the variance, with acceptable values for the goodness-of-fit and goodness-of-prediction values (R^2^ = 0.48 and Q^2^ = 0.39), indicating a good model. The HCA model offers another unsupervised analysis method, with a visual graphical display (Figure 4C) showing two main clusters (I and II). Cluster I encompassed only the green arabica sample (GCA), while the rest of the samples were embedded in cluster II. As the two subclasses, the green canephora specimen was present, alone, in one subclass (A), whereas the roasted authentic samples, along with the Luwak, were grouped in cluster B, revealing the similarity between roasted and Luwak coffees, as revealed by the PCA analysis. Both the PCA score plots and HCA showed the segregation of Luwak coffee toward the roasted samples, with a closer aggregation with the roasted arabica samples (RCA), which is in agreement with the NMR results (Figure 4A). Further examination of the PCA loading plot (Figure 4B) indicated that the phenolic acids and diterpenes were more abundant in the roasted samples than in the Luwak samples. In another attempt to investigate more markers, a supervised OPLS-DA model was established to compare the roasted samples against the Luwak coffee. The supervised model showed the parameters R^2^ and Q^2^ at 0.98 and 0.84, respectively, supporting good model fitness and predictability, and explaining its significant markers with a *p*-value of less than 0.05 (Figure 4D). The OPLS-DA-derived S-plot (Figure 4E) showed other distinctive markers for Luwak, such as the citric acid (L4) and fatty acid series, viz., L20, L22, L23, and L24. On the other hand, hydroxycinnamic acids (caffeoyl, feruloyl, coumaroyl, and dicaffeoyl quinic acids) were more abundant in the roasted *C. arabica* samples. The chlorogenic acid lactones were distinguished as markers for the roasted *C. arabica* samples. These results are aligned with our previously published work on authentic coffee samples [13].

In comparison to the NMR results, both techniques showed good segregation of all samples, suggesting the related composition of both roasted and Luwak coffees, compared to green coffee. However, more markers were detected using the LCMS technique, such as chlorogenic acids and citric acid, which were not revealed using NMR.

### 4.3. SPME-GC/MS Multivariate Data Analysis of Luwak Coffee, Roasted Coffee, and Roasted Coffee with Cardamom

**Figure 5:**
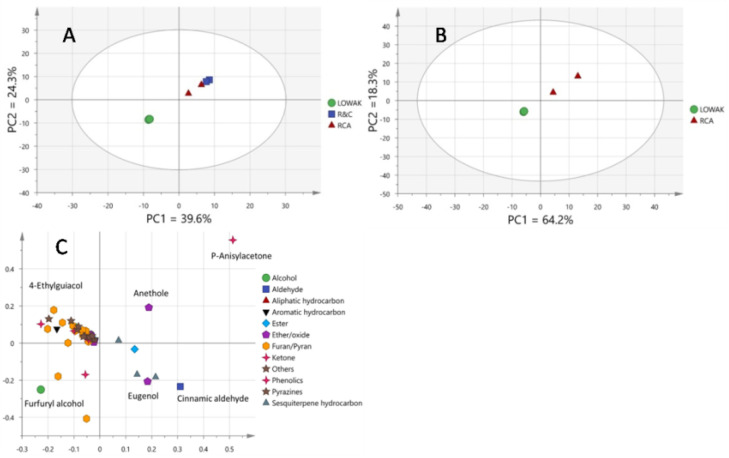
(**A**) PCA model score-plot of the Luwak coffee samples, along with commercially roasted coffee with cardamom and authentically roasted *C. arabica*. (**B**) PCA model score-plot of the Luwak coffee samples, along with authentically roasted *C. arabica*. (**C**) PCA loading plot of the Luwak coffee samples (LUWAK) versus authentically roasted *C. arabica* (RCA).

Multivariate data analysis (MVA) visualized the further differences in the aroma profile of Luwak coffee, compared with those of the RCA and roasted coffee with cardamom. The score plot of the PCA that was derived from all the coffee samples showed two components that accounted for 39.6% and 24.3% of the total variance. The PCA score plot revealed the distinct separation of civet coffee from other roasted coffees (Figure 5A). Civet coffee was found to be clearly separated from the roasted *C. arabica* along the PC1-axis. The PCA model showed high quality in terms of the goodness-of-fit (R^2^X% 0.639). Repeating the model using a subset of RCA versus Luwak coffee showed a higher goodness-of-fit (R^2^X% 0.825), as indicated in Figure 5B. The loading plot of Luwak coffee versus RCA (Figure 5C) showed a higher 4-ethylguiacol level as a potential marker of Luwak coffee, establishing the aroma profiling.

OPLS-DA modeling was further employed to indicate the role of animal fermentation on the aroma profile of roasted coffee. The OPLS-DA score plot of the total data set of Luwak coffee versus that of RCA revealed segregation between the samples on the basis of animal fermentation (see Figure S3A in the Supplementary Materials). Civet coffee and the roasted coffee samples were clearly separated in the predictive component (t [1]). The OPLS-DA model was built with an R^2^Y value of 0.995 and a Q^2^ value of 0.946. The correlation coefficient (R^2^Y) is used to describe how a model fits a set of predicted data sets related to class separation. The high Q^2^ value in the model precludes overfitting. A Q^2^ value of 0.5 is considered to be acceptable for a model derived from biological samples [12].

Permutation tests were performed in the PLS-DA model to confirm the quality of the OPLS-DA model. According to Setoyama et al., if the OPLS-DA model were over-fitted, the R^2^Y and Q^2^ values would not virtually change after permutation [36]. Both parameters were in the range of the requirements for a reliable model; R^2^Y-intercept values fluctuated between 0.0 and 0.795, and the Q^2^-intercept was below 0.05. These values denoted that there was a change in the values of the two parameters (see Figure S3B in the Supplementary Materials).

The OPLS plot showed that the terpenes and esters showed higher abundance, with conventionally roasted coffee showing the most influencing volatiles on the right corner, which belong to terpinyl acetate, *p*-anisylacetone, cinnamic aldehyde, and acetone. In contrast, the S-plot showed that the civet coffee is rich in phenolics alongside furans, with most of the influencing volatiles being 4-ethylguaiacol, furfuryl alcohol, and difurfuryl ether (see Figure S3C in the Supplementary Materials).

This study is the first report on the civet coffee metabolome in comparison with conventional coffee but managed to identify several civet coffee markers. Compared with recent publications, most of the studies focused on analytical techniques by which to determine coffee authenticity for the common types and minimize adulteration [37]. Several markers were reported for the authenticity evaluation of Luwak coffee, such as caffeine, inositol, and pyroglutamic acid, by using GC-MS [38] obtained from the robusta Luwak coffee, while citric acid, malic acid, and glycolic acid were reported to be characteristic of arabica Luwak coffee [39]. Citric acid and malic acid, as compound markers of Luwak coffee [2], appeared in alignment with those in the NMR spectra evaluation in this study. Likewise, our results revealed a higher abundance of furans, pyridine, and pyrazine derivatives in the Luwak coffee post-roasting tests, compared with unfermented roasted coffee and in accordance with the previous report [40]. Moreover, although elaidic acid was previously reported in arabica coffee pulp and husk [41], our research is the first to report it in Luwak coffee. With regard to the study limitations, the current research examined civet coffee from one commercial source that has yet to be distinguished from other specimens. Further comparisons between the same coffee used in Luwak coffee prior to the fermentation step should aid in dissecting the impact of this step on the Luwak coffee metabolome as the analyzed coffee proved to have been subjected to both fermentation and roasting processes, as is evident from the presence of furan compounds. The administration of different coffee types to the civet animal and the monitoring of changes in metabolome using the same approach as that described herein should aid in identifying the best sources for producing this premium type of coffee.

Future biological studies are recommended for revealing civet coffee’s effects, especially in terms of targeting CNS compared to other coffee types. They will aid in correlating the metabolome composition to achieve certain targeted effects.

## 5. Conclusions

Three different technology platforms were employed for Luwak coffee classification, including NMR, LC-MS, and SPME/GC-MS, to show the significant classifications among all samples and aid in identifying potential novel markers to distinguish Luwak coffee from other coffee samples. The markers indicated that *C. arabica* was the source of Luwak coffee. The roasting process that was applied to Luwak coffee and roasted *C. arabica* preparation had a pivotal role in their comparable metabolite profile similarity and their distance from the green coffee samples, as revealed from the NMR and LC-MS models. The Luwak coffee metabolite markers revealed by the NMR included elaidic acid, kahweol, and di-furfuryl ether. The latter was also identified as a marker for Luwak coffee, using SPME/GC-MS analysis. This study also confirmed the impact of the fermentation step prior to roasting on the aroma profile by using SPME coupled with GC/MS, as exemplified by the higher abundance of guaiacol derivatives, pyrazines, and furans in roasted Luwak coffee compared with roasted *C. arabica*. Finally, such a comparative metabolomics approach overcomes the limitation of detection by using one technique versus another. For example, some metabolite markers, such as citric acid, were identified in the LC-MS versus the elaidic and other fatty acids in NMR via other markers, i.e., di-furfuryl ether was detected in both NMR and SPME/GC-MS. Such a comparative metabolomics approach can be used for the quality control assessment of other distinctive or premium coffee products from regular ones in the future. A comparison of Luwak’s health benefits compared to roasted coffee should also follow, based on these findings, as revealed using metabolomics.

## Figures and Tables

**Table 1:** Resonance assignments with the chemical shifts of constituents identified in 600 MHz ^1^H NMR, heteronuclear single-quantum correlation spectroscopy (HSQC), and the heteronuclear multiple-bond coherence (HMBC) spectra of Luwak coffee (methanol d4; CD_3_OD).

No	Metabolite	Assignment	^1^H (Multiplicity)	HSQC	HMBC
1	(*E*)-octadec-9-enoic acid	H-9	5.33	129	C-7 (27.9)
		H-10	5.33	129	C-12 (27.9)
		H-2	2.35	34.8	C-1 (174.6), C-3 (26.4)
		H-8, H-11	2.3	34.8	
		H-3	1.6	25.9	C-1 (174.6)
2	Trigonelline	H-2	9.20 s	147.5	
		H-4	8.89 d	146.8	C-5 (129.2), C-2 (146.8)
		H-5	8.09 t	129.2	C-3 (143.9)
		H-6	8.86 d	146.8	
		CH3	4.43 s	48.8	C-2 (147.5)
3	Caffeoyl shikimic acid	CH_2_-2axial	2.09	40.4	
		CH_2_-2 equatorial	2	40.4	
		H-5	5.26	70.5	C-4 (74.8)
		H-8′	6.34	109.5	
		H-7′	7.56	146.9	
		H-2′	7.04	115	
		H-5′	6.77	116.4	
		H-6′	6.95	122.8	
4	Caffeine	H-8	3.35	28.1	C-1 (156.8), C-2 (153.3)
		H-7	3.53	30	C-2 (153.3), C-3 (149.8)
		H-6	3.97	33.7	C-4 (108.8), C-5 (144.0)
		H-5	7.86	144.1	C-2 (115.0)
5	Kahweol	H-1	5.95 d (10.2)	139.6	C-5 (45.7), C-9 (49.7), C-10 (42.9)
		H-2	6.21 d (10.2)	116.2	C-10 (42.9)
		H-3	---	151.5	
		H-4	---	122.9	
		H-5	2.57	45.7	
		H-9	1.59	49.7	
		H-10	---	42.9	
		H-18	6.21 (10.2)	109	C-3 (151.5), C-4 (122.9)
		H-19	7.23	141.8	C-3 (151.5), C-4 (122.9)
		H-20	0.97	15.9	C-1 (156.8), C-5 (45.7), C-9 (49.7), C-10 (42.9)
6	Caffeic acid	H-2	7.06 d	115	
		H-5	6.77 d	116.3	
		H-6	6.98 dd	124.3	
		H-7	7.57 d	146.8	
		H-8	6.28 d	117.1	
7	*N*-methylpyridinium	H-2, H-6	9.90 d	146.8	
		H-3, H-5	8.05 t	128.4	
		H-4	8.57 t	146.5	C-2 (146.8), C-6 (146.8)
		CH3	4.42 s	48.8	C-2 (146.8), C-6 (146.8)
8	Myo-inositol	H	3.41 dd	73.1	73.1
9	Xanthine	H-8	7.93 s	132.9	
10	Difurfuryl ether	H-3	6.26	115.4	
		H-4	6.29	115.3	
		H-5	7.57	14.1	
11	fumaric acid	H	6.61	132.6	C=O (173.5)
12	Caffeine	H-8	3.35	28.1	C-1 (156.7), C-2 (153.3)
		H-7	3.53	30	C-2 (153.3), C-3 (149.8)
		H-6	3.97	33.7	C-4 (108.8), C-5 (144.0)
		H-5	7.86	144.1	C-2 (108.8)
13	Lactic acid	CH_3_	1.34	23.5	
		CH	4	68.2	
14	Sucrose	H-1	5.37	93.8	C-5 (74.5), C-1′ (105.3)
		H-2	3.41	73.1	C-3 (74.4)
		H-3	3.68	74.4	
		H-4	3.34	71.4	C-3 (74.4), C-5 (74.5)
		H-5	3.81	74.5	
		H-6	3.68	61.7	
		H-2′	4.08	79.3	
		H-3′	3.95	74.1	C-1′ (105.3), C-5′ (64.5), C-2′ (79.3)
		H-4′	3.75	83.8	
		H-5′	3.7	64.5	
		H-6′	3.75	63.2	

**Table 2:** Metabolites identified in the methanol extract of Luwak coffee via UPLC-PDA-ESI-HRMS in negative mode.

No	RT (min)	Compound Name	Chemical Class	[M-H]-	Molecular Formula	Mass Error	MS/MS Fragments	References
L1	0.27	Malic acid	Organic acid	133.01411	C_4_H_5_O_5_−	−2.36	n.d	[13]
L2	0.22	*O*-Malonyl-hexopyranoside	Sugar	471.07425	C_19_H_19_O_14_^−^	4.44	n.d	[13]
L3	0.32	Di-*O*-hexoside	Sugar	341.10815	C_12_H_21_O_11_^−^	−2.32	179,161	[13]
L4	0.37	Citric acid	Organic acid	191.01926	C_6_H_7_O_7_^−^	−2.43	111,173	[13]
L5	0.38	Quinic acid	Organic acid	191.05556	C_7_H_11_O_6_^−^	−2.86	173, 111	[13]
L6	0.38	*O*-Caffeoylquinic acid	Phenolic acid	353.08664	C_16_H_17_O_9_^−^	−3.37	191,179,135	[13]
L7	3.2	Caffeoylshikimic acid	Phenolic acid	335.07614	C_16_H_15_O_8_^−^	−3.21	179,161,135	[13]
L8	6.1	Dicaffeoylquinic acid	Phenolic acid	515.11804	C_25_H_23_O_12_^−^	−2.82	353,335	[13]
L9	6.3	Feruloylquinic acid	Phenolic acid	367.10223	C_17_H_19_O_9_^−^	−3.33	161,193,135	[13]
L10	6.6	P-Coumaroyl quinic acid	Phenolic acid	337.09174	C_16_H_17_O_8_^−^	−3.41	191,163	[13]
L11	7.01	Atractyligenin-O-hexoside	Diterpene	481.24234	C_25_H_37_O_9_^−^	−4.08	301	[13]
L12	7.11	Carboxtatractyligenin-O-hexoside	Diterpene	525.23193	C_26_H_37_O_11_^−^	−4.18	396,203	[13]
L13	7.7	Trihydroxy-kauranoic acid	Diterpene	351.21664	C_20_H_31_O_5_^−^	−3.12	289,321	[13]
L14	7.7	Caffeoyl-feuloylquinic acid	Phenolic acid	529.13293	C_26_H_25_O_12_^−^	−2.35	367,353	[13]
L15	7.9	Desoxycarboxyatractyligenin-O-hexoside	Diterpene	771.34113	C_37_H_55_O_17_−	−4.31	727	[13]
L16	7.9	Desoxyatractyligenin-O-hexoside	Diterpene	727.35138	C_36_H_55_O_15_^−^	−4.42	643,625	[13]
L17	8.1	Caffeoyl-N-tryptophan	Amino acid	365.11272	C_20_H_17_N_2_O_5_^−^	−2.08	135,229	[13]
L18	8.3	Unknown chlorogenic acid	Phenolic acid	437.14359	C_21_H_25_O_10_^−^	−3.95	173,275	[13]
L19	8.47	Di-caffeoylquinolactone	Phenolic acid	497.10716	C_25_H_22_O_11_^−^	−2.25	335	[13]
L20	8.56	Unknown fatty acid	Fatty acid	538.23956	C_25_H_48_O_11_N^−^	−4.25	311,198,180	[13]
L21	8.8	Isovaleryl-atractyligenin-O-hexoside	Diterpene	565.29987	C_30_H_45_O_10_^−^	−3.45	481,463,303	[13]
L22	12.1	Unknown fatty acid	Fatty acid	311.12355	C_20_H_13_O_3_^−^	−3.12	183	[13]
L23	12.5	Unknown fatty acid	Fatty acid	325.18341	C_14_H_29_O_8_^−^	−9.61	183	[13]
L24	13.7	Unknown fatty acid	Fatty acid	339.19907	C_15_H_31_O_8_^−^	−9.91	183	[13]

**Table 3:** The volatiles detected using SPME/GC-MS in Luwak coffee, alongside roasted coffee (RCA) and roasted coffee with cardamom, expressed as a relative percentile (mean ± SD).

Category	Metabolite	RI	RT	Luwak Samples	Roasted Coffee with Cardamom (R&C)	Roasted *C. arabica* (RCA)
Alcohol	1-Octanol	1101.5	10.3042		0.29 ± 0.251	
	2,7-dimethyl-4-Octene-2,7-diol	1152.7	11.0333		0.2 ± 0.09	
	Cineole	1007.2	8.8292	0.35 ± 0.433	1.98 ± 1.405	
	Furfuryl alcohol	842.7	5.7867	12.1 ± 2.204		5.5 ± 7.774
	Nerolidol	1519.7	15.7508		3.71 ± 4.156	
	Maltol	1115.9	10.5092	2.23 ± 2.078		
Aldehyde	2-Methoxy-4-methylbenzaldehyde	1132.7	10.7492	0.41 ± 0.361		
	Cinnamic aldehyde	1264.8	12.5617		1.44 ± 2.038	14.54 ± 0.247
Aliphatic hydrocarbon	Dodecane	1163.5	11.1875	0.17 ± 0.161		
	Hexadecane	1545.4	16.1208	0.18 ± 0.184	0.01 ± 0.017	
	Pentadecane	1450.6	14.8658		0.44 ± 0.372	
	Tetradecane	1356.1	13.7125	0.49 ± 0.406		
Aromatic hydrocarbon	Naphthalene	1169.6	11.275	4.73 ± 1.833		
	Styrene	867.9	6.31	0.09 ± 0.093		
Ester	Decyl acetate	1369.9	13.8808		0.94 ± 0.957	0.13 ± 0.186
	Diisobutyl phthalate	1812.3	20.935	0.09 ± 0.083	0.29 ± 0.332	
	Ethylene diacetate	851.8	5.9767	0.88 ± 0.802		
	Linalyl acetate	1216.4	11.9242		12.16 ± 10.269	2.32 ± 0.707
	Myrcenylacetate	1285.3	12.8317		0.33 ± 0.262	
	Oxalic acid, allyl isobutyl ester	1037.2	9.3	0.07 ± 0.063		
	Terpinyl acetate	1316.9	13.2325		35.53 ± 49.328	13.05 ± 18.453
Ether/oxide	Anethole	1267.7	12.5992			3.98 ± 5.211
	Biphenyl oxide	1382.4	14.035	0.12 ± 0.116		
	Cineol	1007.2	8.8292	0.35 ± 0.433	1.98 ± 1.405	
	Dicyclobutylidene oxide	1083.4	10.0233	0.61 ± 0.291		
	Eugenol	1334.8	13.4517		3.44 ± 3.801	5.84 ± 0.965
Furan / pyrrole	2-Furanmethanol	842.7	5.7867	6.05 ± 1.102		2.75 ± 3.887
	Acetylfuran	891.6	6.8008	0.65 ± 0.048		
	Furfural	820.3	5.3225	4.18 ± 1.87		4.55 ± 6.44
	Furfuryl 3-methylbutanoate	1190.2	11.5683	2.06 ± 1.164		
	Furfuryl acetate	969.5	8.185	6.47 ± 4.393		
	2-Furfuryl-5-methylfuran	1149.8	10.9933	3.42 ± 0.901		
	2-Furfurylfuran	1057.5	9.6175	0.73 ± 0.694		
	2-Pentylfuran	963.1	8.0742	1.04 ± 0.909		
	5-Methyl furfural	947.4	7.8008	2.56 ± 0.609		0.44 ± 0.626
	Difurfuryl ether	1274.8	12.6925	8.96 ± 6.84		
	1-Furfurylpyrrole	1157.4	11.1008	1.44 ± 0.383		
	2-Formyl-1-methylpyrrole	990.1	8.5442	0.65 ± 0.609		
	2-Formyl-4,5-dimethyl-pyrrole	1141.3	10.8717	0.45 ± 0.425		
	*N*-Furfurylpyrrole	1157.4	11.1008	1.44 ± 0.383		
Ketone	2,2-Dimethylbutanone	943.3	7.73	0.13 ± 0.132		
	3,3-dimethyl-2-butanone	1550.9	16.2008	0.4 ± 0.257		
	*p*-Anisylacetone	1374.1	13.9325		0.2 ± 0.285	29.78 ± 40.553
Monoterpene hydrocarbon	alpha-Terpineol	1175.5	11.3583		5.69 ± 5.262	
	Camphor	1130.3	10.7142		0.23 ± 0.127	
	Isoterpinolene	1061.3	9.6775		0.6 ± 0.103	
	Terpin-4-ol	1161	11.1517		0.95 ± 0.754	
	Unknown monoterpene	948.2	7.815		0.18 ± 0.105	
Others	Butyryl lactone	895.6	6.8833	2.74 ± 2.129		
Phenolics	4-Ethylguaiacol	1256	12.445	9.59 ± 4.57		
	4-Vinylguaiacol	1302.3	13.0533	1.78 ± 1.197		1.28 ± 1.812
	o-Guaiacol	1070.7	9.8242	1.52 ± 0.078		
Pyrazines	1-(6-Methyl-2-pyrazinyl)-1-ethanone	1097.4	10.2417	0.28 ± 0.262		
	2,3-dimethylpyrazine	899.9	6.9725	0.1 ± 0.099		
	2,6-dimethylpyrazine	894.8	6.8667	1.62 ± 1.205		
	2-Acetyl-3-methylpyrazine	1097.4	10.2417	0.21 ± 0.27		
	2-Ethyl-3-methylpyrazine	978.6	8.345	1.05 ± 1.181		
	2-Methyl-3,5-diethylpyrazine	1130.5	10.7175	0.56 ± 0.115		
	2-Methyl-5-propenyl-pyrazine	1181.4	11.4433	0.68 ± 0.248		
	5-Methyl-2,3-diethylpyrazine	1126.1	10.655	0.25 ± 0.063		
	5-Methyl-5H-cyclopenta [b]pyrazine	1123.5	10.6183	0.23 ± 0.235		
	Methylpyrazine	842.7	5.7867	6.05 ± 1.102		2.75 ± 3.887
	Pyrazine, 2-ethyl-3-methyl-	978.6	8.345	1.62 ± 1.404		
	2-Methyl-5-(1-propenyl)-, (E)-pyrazine	1181.4	11.4433	0.68 ± 0.248		
Sesquiterpene hydrocarbon	alpha-Farnesene	1461.1	14.9942		1.02 ± 1.11	
	beta-Curcumene	1471.7	15.1225			3.68 ± 0.702
	beta-Eudesmene	1463.6	15.0242		1.19 ± 0.518	
	Calamenene	1493.1	15.3833		0.17 ± 0.151	
	beta-Caryophyllene	1395.9	14.1992		2.09 ± 1.751	
	Curcumene	1444.6	14.7933			7.28 ± 0.257
	Germacrene	1484.4	15.2775		6.25 ± 5.915	
	alpha-Humulene	1430.2	14.6175		0.45 ± 0.31	
	alpha-Bergamotene	1400.2	14.2525		2.94 ± 3.195	
	beta-Farnesene	1411.8	14.3933			0.63 ± 0.386

## Data Availability

The data presented in this study are available in article and supplementary material.

## Supplementary Materials

The following supporting information can be downloaded at: https://www.mdpi.com/article/10.3390/metabo13020173/s1, Figure S1: (A) HSQC NMR spectra for the assignment of 1, sucrose; 2, 5-CQA; 3, 4-CQA; 4, trigonelline; 5, caffeine; 6, quinic acid (B) HSQC NMR spectra for the assignment of caffeoyl shikimic acid (C, D) HSQC and HMBC NMR spectra for assignment of octadec-9-enoic acid as a major fatty acid constituent in Luwak coffee seeds. (E) HSQC NMR spectra for the assignment of nitrogen compounds (caffeine and trigonelline) (F) HMBC NMR spectra for the assignment of n-methylpyridinium. All assignment details are listed in Table 1. Figure S2: Percentile proportion of metabolite groups in different coffee samples as identified by SPME/GC-MS; Figure S3: (A) OPLS-DA model score plot of Luwak coffee versus roasted coffee. (B) Permutation analysis to validate the OPLS-DA model score plot of Luwak coffee versus roasted coffee. (C) OPLS-DA model S-plot of Luwak coffee versus roasted coffee.

## References

[1] Jumhawan U., Putri S.P., Yusianto, Marwani E., Bamba T., Fukusaki E. (2013). Selection of discriminant markers for authentication of asian palm civet coffee (kopi luwak): A metabolomics approach. J. Agric. Food Chem..

[2] Jumhawan U., Putri S., Yusianto, Bamba T., Fukusaki E. (2016). Quantification of coffee blends for authentication of asian palm civet coffee (kopi luwak) via metabolomics: A proof of concept. J. Biosci. Bioeng..

[3] Muzaifa M., Hasni D., Rahmi F. (2019). What is kopi luwak? A literature review on production, quality and problems. IOP Conf. Ser. Earth and Environ. Sci..

[4] Marcone M. (2004). Composition and properties of indonesian palm civet coffee (kopi luwak) and ethiopian civet coffee. Food Res. Int..

[5] Lachenmeier D., Schwarz S. (2021). Digested civet coffee beans (kopi luwak)—an unfortunate trend in specialty coffee caused by mislabeling of coffea liberica?. Foods.

[6] Iswanto T., Shovitri M., Altway A., Widjaja T., Kusumawati D.I., Lisdyanti P. (2019). Isolation and identification of caffeine-degrading bacteria from soil, coffee pulp waste and excreted coffee bean in luwak feces. Feces.

[7] Watanabe H., Ng C.H., Limviphuvadh V., Suzuki S., Yamada T.J.P. (2020). Gluconobacter dominates the gut microbiome of the asian palm civet paradoxurus hermaphroditus that produces kopi luwak. luwak.

[8] Aditiawati P., Astuti D.I., Kriswantoro J.A., Khanza S.M., Kamarisima, Irifune T., Amalia F., Fukusaki E., Putri S.P. (2020). Gc/ms-based metabolic profiling for the evaluation of solid state fermentation to improve quality of arabica coffee beans. Metabolomics.

[9] Foudah A.I., Alam P., Abdel-Kader M.S., Shakeel F., Alqasoumi S.I., Salkini A.M., Yusufoglu H.S. (2020). High-performance thin-layer chromatographic determination of trigonelline content in various extracts and different varieties of some commercial coffees available in the saudi arabian market. JPC–J. Planar Chromatogr.–Modern TLC.

[10] Foudah A.I., Shakeel F., Salkini M.A., Alshehri S., Ghoneim M.M., Alam P. (2022). A green high-performance thin-layer chromatography method for the determination of caffeine in commercial energy drinks and formulations. Materials.

[11] Alam P., Shakeel F., Ali A., Alqarni M.H., Foudah A.I., Aljarba T.M., Alkholifi F.K., Alshehri S., Ghoneim M.M., Ali A. (2022). Simultaneous determination of caffeine and paracetamol in commercial formulations using greener normal-phase and reversed-phase hptlc methods: A contrast of validation parameters. Molecules.

[12] Jumhawan U., Putri S.P., Bamba T., Fukusaki E. (2015). Application of gas chromatography/flame ionization detector-based metabolite fingerprinting for authentication of asian palm civet coffee (kopi luwak). J. biosci. bioeng..

[13] El-Hawary E.A., Zayed A., Laub A., Modolo L.V., Wessjohann L., Farag M.A. (2022). How does lc/ms compare to uv in coffee authentication and determination of antioxidant effects? Brazilian and middle eastern coffee as case studies. Antioxidants.

[14] Abdelwareth A., Zayed A., Farag M.A. (2021). Chemometrics-based aroma profiling for revealing origin, roasting indices, and brewing method in coffee seeds and its commercial blends in the middle east. Food Chem..

[15] Porzel A., Farag M.A., Mülbradt J., Wessjohann L.A. (2014). Metabolite profiling and fingerprinting of hypericum species: A comparison of ms and nmr metabolomics. Metabolomics.

[16] Farag M.A., Porzel A., Mahrous E.A., El-Massry M.M., Wessjohann L.A. (2015). Integrated comparative metabolite profiling via ms and nmr techniques for senna drug quality control analysis. Anal. Bioanal. Chem..

[17] Farag M.A., Rasheed D.M., Kamal I.M. (2015). Volatiles and primary metabolites profiling in two hibiscus sabdariffa (roselle) cultivars via headspace spme-gc-ms and chemometrics. Food Res. Int..

[18] Farag M.A., Hegazi N., Dokhalahy E., Khattab A.R. (2020). Chemometrics based gc-ms aroma profiling for revealing freshness, origin and roasting indices in saffron spice and its adulteration. Food Chem..

[19] Zayed A., Abdelwareth A., Mohamed T.A., Fahmy H.A., Porzel A., Wessjohann L.A., Farag M.A. (2022). Dissecting coffee seeds metabolome in context of genotype, roasting degree, and blending in the middle east using nmr and gc/ms techniques. Food Chem..

[20] Farag M.A., Porzel A., Wessjohann L.A. (2015). Unraveling the active hypoglycemic agent trigonelline in balanites aegyptiaca date fruit using metabolite fingerprinting by nmr. J. Pharm. Biomed. Anal..

[21] del Campo G., Berregi I., Caracena R., Zuriarrain J. (2010). Quantitative determination of caffeine, formic acid, trigonelline and 5-(hydroxymethyl) furfural in soluble coffees by 1h nmr spectrometry. Talanta.

[22] Riedel A., Hochkogler C.M., Lang R., Bytof G., Lantz I., Hofmann T., Somoza V. (2014). N-methylpyridinium, a degradation product of trigonelline upon coffee roasting, stimulates respiratory activity and promotes glucose utilization in hepg2 cells. Food Funct..

[23] Kotyczka C., Boettler U., Lang R., Stiebitz H., Bytof G., Lantz I., Hofmann T., Marko D., Somoza V. (2011). Dark roast coffee is more effective than light roast coffee in reducing body weight, and in restoring red blood cell vitamin e and glutathione concentrations in healthy volunteers. Mol. Nutr. Food Res..

[24] Belton P.S., Colquhoun I.J., Kemsley E.K., Delgadillo I., Roma P., Dennis M.J., Sharman M., Holmes E., Nicholson J.K., Spraul M. (1998). Application of chemometrics to the 1h nmr spectra of apple juices: Discrimination between apple varieties. Food Chem..

[25] Girelli C.R., Angilè F., Del Coco L., Migoni D., Zampella L., Marcelletti S., Cristella N., Marangi P., Scortichini M., Fanizzi F.P. (2019). ^1^H-NMR Metabolite Fingerprinting Analysis Reveals a Disease Biomarker and a Field Treatment Response in *Xylella fastidiosa* subsp. *pauca*-Infected Olive Trees. Plants.

[26] Wei F., Furihata K., Hu F., Miyakawa T., Tanokura M. (2010). Complex mixture analysis of organic compounds in green coffee bean extract by two-dimensional nmr spectroscopy. Magn. Reson. Chem..

[27] Hall L. (1964). The conformations of cyclic compounds in solution. I. Shikimic acid. J. Org. Chem..

[28] Alexandri E., Ahmed R., Siddiqui H., Choudhary M.I., Tsiafoulis C.G., Gerothanassis I.P. (2017). High resolution nmr spectroscopy as a structural and analytical tool for unsaturated lipids in solution. Molecules.

[29] Luisa P.F., Flavio M.B., Fabiana C.R., Gerson S.G., Jose H.d.S.T., Marcelo R.M. (2015). Fatty acid profiles and parameters of quality of specialty coffees produced in different brazilian regions. Afr. J. Agric. Res..

[30] Yang S., Hao Y., Wang J., Wang H., Zheng Y., Tian H., Liu Y., Sun B. (2017). Selective catalytic dehydration of furfuryl alcohol to 2, 2′-difurfuryl ether using a polyoxometalate catalyst. Sci. Rep..

[31] Karunanithi G., Varadappan A.M.S. (2022). Exploring the effectiveness of novel coffea arabica leaf pigment as a natural antioxidant additive for date seed biodiesel. Fuel.

[32] Fang N., Yu S., Prior R.L. (2002). Lc/ms/ms characterization of phenolic constituents in dried plums. J. Agric. Food Chem..

[33] Asamenew G., Kim H.W., Lee M.K., Lee S.H., Lee S., Cha Y.S., Lee S.H., Yoo S.M., Kim J.B. (2019). Comprehensive characterization of hydroxycinnamoyl derivatives in green and roasted coffee beans: A new group of methyl hydroxycinnamoyl quinate. Food Chem. X.

[34] Bianco G., Abate S., Labella C., Cataldi T.R. (2009). Identification and fragmentation pathways of caffeine metabolites in urine samples via liquid chromatography with positive electrospray ionization coupled to a hybrid quadrupole linear ion trap (ltq) and fourier transform ion cyclotron resonance mass spectrometry and tandem mass spectrometry. Rapid Commun. Mass Spectro. RCM.

[35] Lee L.W., Cheong M.W., Curran P., Yu B., Liu S.Q. (2016). Modulation of coffee aroma via the fermentation of green coffee beans with rhizopus oligosporus: Ii. Effects of different roast levels. Food Chem..

[36] Setoyama D., Iwasa K., Seta H., Shimizu H., Fujimura Y., Miura D., Wariishi H., Nagai C., Nakahara K. (2013). High-throughput metabolic profiling of diverse green coffea arabica beans identified tryptophan as a universal discrimination factor for immature beans. PLoS ONE.

[37] Burns D.T., Walker M.J. (2020). Critical review of analytical and bioanalytical verification of the authenticity of coffee. J. AOAC Int..

[38] Medina S., Pereira J.A., Silva P., Perestrelo R., Câmara J.S. (2019). Food fingerprints—A valuable tool to monitor food authenticity and safety. Food Chem..

[39] Putri S.P., Ikram M.M.M., Sato A., Dahlan H.A., Rahmawati D., Ohto Y., Fukusaki E. (2022). Application of gas chromatography-mass spectrometry-based metabolomics in food science and technology. J. Biosci. Bioeng..

[40] Kim S.-J., Lee S., Bang E., Lee S., Rhee J.-K., Na Y.-C. (2019). Comparative evaluation of flavor compounds in fermented green and roasted coffee beans by solid phase microextraction-gas chromatography/mass spectrometry. Flavour Fragr. J..

[41] Lestari W., Hasballah K., Listiawan M., Sofia S. (2022). Identification of antioxidant components of gayo arabica coffee cascara using the gc-ms method. IOP Conf. Ser. Earth and Environ. Sci..

