# Supplementary material for: Metabolite Profiling of Premium Civet Luwak Bio-Transformed Coffee Compared with Conventional Coffee Types, as Analyzed Using Chemometric Tools
Source: Metabolites. 2023 Jan 24;13(2):173. doi: 10.3390/metabo13020173 (PMC9960232; doi:10.3390/metabo13020173)
Supplement: Supplementary file 1 [file metabolites-13-00173-s001.zip › metabolites-2166538-supplementary.pdf]

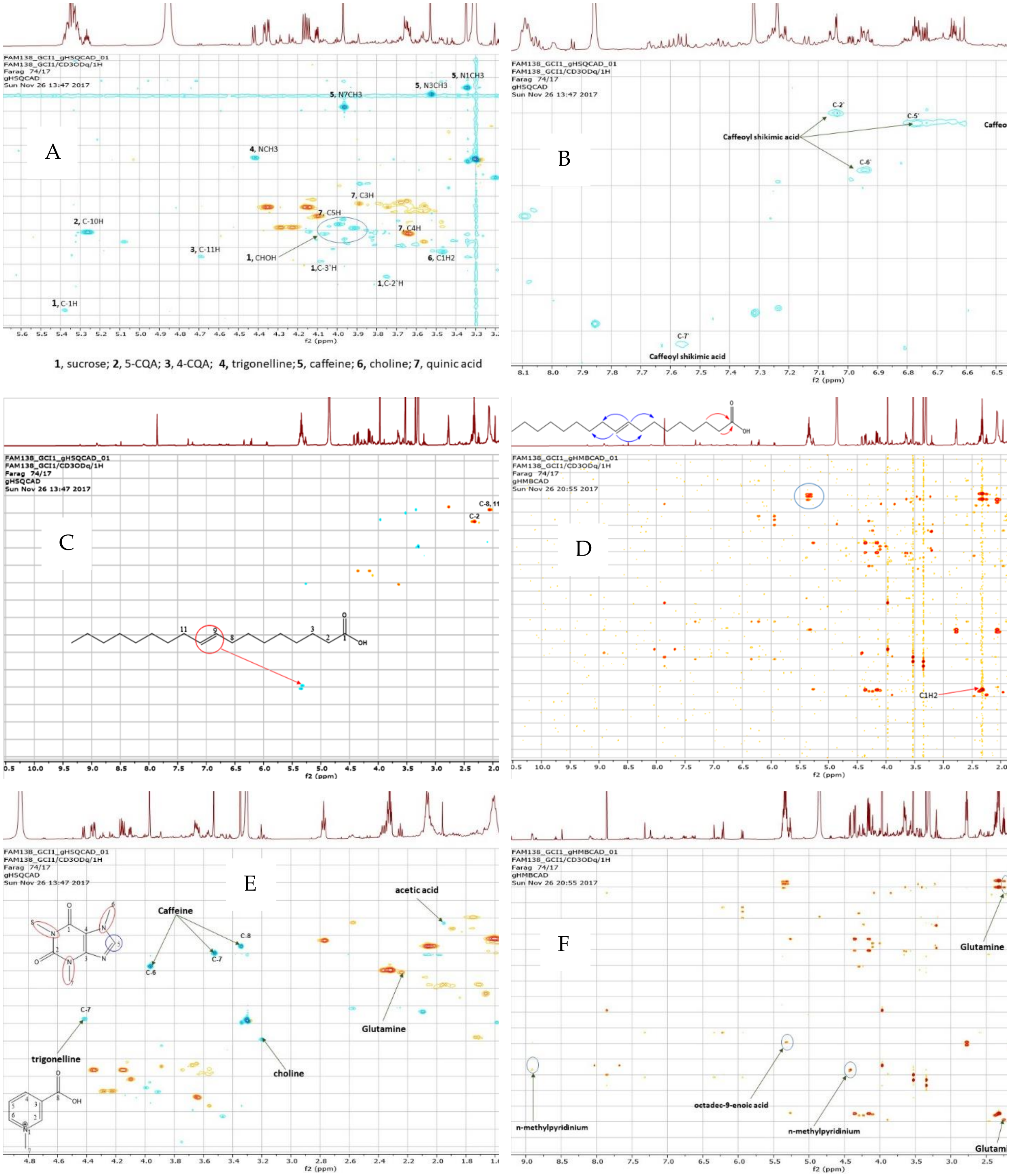

Suppl. Figure S1 (A) HSQC NMR spectra for assignment of 1, sucrose; 2, 5-CQA; 3, 4-CQA; 4, trigonelline; 5, caffeine; 6, quinic acid (B) HSQC NMR spectra for assignment of caffeoyl shikimic acid (C, D) HSQC and HMBC NMR spectra for assignment of octadec-9-enoic acid as a major fatty acid constituent in Luwak coffee seeds. (E) HSQC NMR spectra for assignment of Nitrogen compounds (Caffeine and

trigonelline) (F) HMBC NMR spectra for assignment of n-methylpyridinium. All assignment details are listed in Table 1

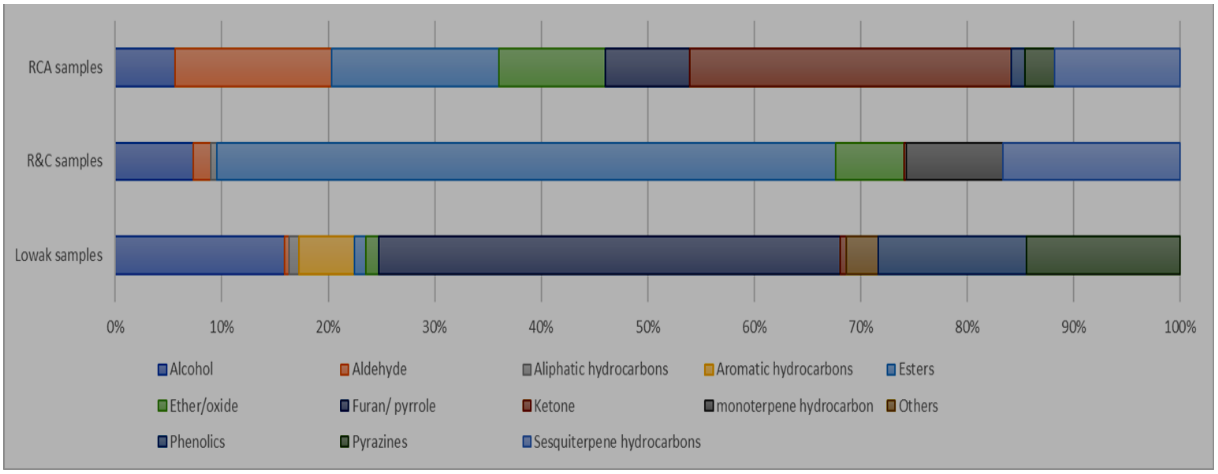

Suppl. Figure S2 Percentile proportion of metabolite groups in different coffee samples as identified by SPME/GC-MS

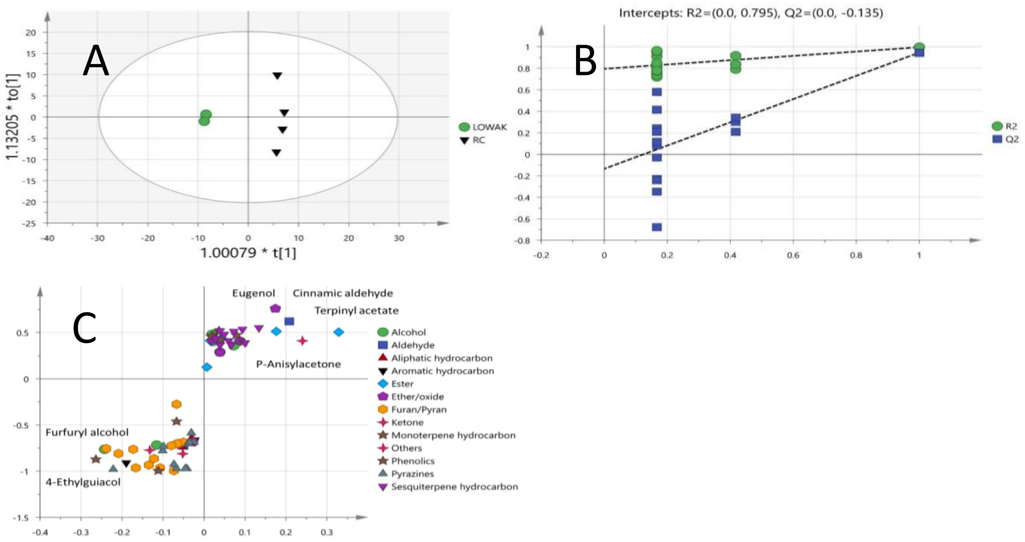

Suppl. Figure S3 (A) OPLS-DA model score plot of Luwak coffee versus roasted coffee, (B)Permutation analysis to validate OPLS-DA model score plot of Luwak coffee versus roasted coffee, (C) OPLS-DA model S-plot of Luwak coffee versus roasted coffee
